# General practice for the poor and specialist services for the rich: inequality evidence from a cross-sectional survey on Hangzhou residents, China

**DOI:** 10.1186/s12939-019-0966-6

**Published:** 2019-05-14

**Authors:** Tao Zhang, Chaojie Liu, Lingrui Liu, Yong Gan, Wei Lu, Hongbing Tao

**Affiliations:** 1 Department of Health Management, School of Medicine and Health Management, Tongji Medical College, Huazhong University of Science and Technology, No.13, Aviation Road, Qiaokou District, Wuhan, Hubei China; 2 School of Psychology and Public Health, La Trobe University, Melbourne, Australia; 3 Department of Health Policy and Management, Yale School of Public Health, New Haven, CT USA; 4 Global Health Leadership Initiative, Yale University, New Haven, CT USA; 5 Department of Social Medicine and Health Management, School of Public Health, Tongji Medical College, Huazhong University of Science and Technology, Wuhan, Hubei Province China

**Keywords:** Inequality, Concentration index, General practice, Specialist care, China

## Abstract

**Background:**

Inequalities in health care services are becoming an increasing concern in the world including in China. This study measured the income-related inequalities of residents in Hangzhou of China in access to general practice and specialist care and identified socioeconomic factors associated with such inequalities.

**Methods:**

A cross-sectional questionnaire survey was conducted on 1048 residents in ten urban communities in Hangzhou, China. The percentage and frequency of respondents visiting general practice (GP) and hospital specialist clinics over the past four weeks prior to the survey were estimated. Income-related inequalities in access to these services were measured by the concentration index. Logistic regression and Poisson regression models were established to decompose the contributions of socioeconomic factors (residency, income, education, marital status, and social health insurance) to the inequalities in the probability and frequency of accessing these services, respectively, after adjustment for the needs factors (age, sex and illness conditions).

**Results:**

The GP services were in favor of the poor, with a concentration index of − 0.0464 and − 0.1346 for the probability and frequency of GP visits, respectively. In contrast, the specialist services were in favor of the rich, with a concentration index of 0.1258 and 0.1279 for the probability and frequency of specialist visits, respectively. Income is the biggest contributor to the inequalities, except for the frequency of visits to specialists in which education played the greatest role.

**Conclusions:**

Income-related inequalities in GP and specialist care are evident in China. Policy interventions should pay increasing attention to the emergence of a two-tier system, potentially enlarging socioeconomic disparities in health care services.

**Electronic supplementary material:**

The online version of this article (10.1186/s12939-019-0966-6) contains supplementary material, which is available to authorized users.

## Background

Universal health coverage endorsed by the World Health Organisation (WHO) calls for equal access to health care services for people who need them regardless of their socioeconomic status (SES). However, many health systems in the world are facing serious challenges in equity of care provision [1]. China is no exception. Despite great success in the past few decades in economic development and the achievement of universal coverage of health insurance in recent years, inequalities in health care services have remained a serious issue of concern in China [2–4]. Previous studies demonstrate that the urban-rural disparities in health services have seriously jeopardised the population health outcomes of China [5, 6]. As a result, the most recent round of health reform launched in 2009 considers equity as one of the core policy goals of the reform [7].

The Chinese government has attempted to address the inequity problem through equalising primary care in the community and encouraging hospitals to focus on acute specialist care. This is supposed to ensure equalities in essential medical and public health services under limited investment and resources and pave a pathway towards universal health coverage for the future [8]. However, consumers in China enjoy freedom to choose care providers. Pro-rich inequalities existed in healthcare services utilization, especially in inpatient service [9]. Meanwhile, empirical evidence shows that high quality resources have been concentrated in large hospitals, which make them more attractive to those with a higher income [10]. These wealthier people tend to bypass primary care and seek medical attention directly from hospital specialists even for minor illness conditions. In addition, there is a lack of trust in primary care delivered by general practitioners (GP) in community health services, thus resulting in more people prefer tertiary hospitals [11].

The concept of GP emerged in late 1990s in China, with an expectation to replace “barefoot doctors” (lay health workers with limited vocational training), a legacy of Chairman Mao’s era as a temporary measurement to cope with the serious shortage of health workforce. Unfortunately, most GPs in China have continued to have a lower level of education compared with their hospital counterparts.

In Chinese health system, GP services and specialist care are given different functions and positioning. GPs in community health centers, as health gatekeepers, mainly provide primary healthcare focusing on minor and chronic diseases and referral to hospitals for more complex problems. Specialist care is supposed to pay more attention to patients with serious illness. However, most of patients did not reasonably choose health providers according to the severity of their diseases [10].

In order to encourage people to seek GP services in the community, the government in China developed several policies. For example, residents are encouraged to sign a contract with their local GPs in exchange for some free services and entitlements, such as quick appointments and referral to specialists [12–14]. The government approved price level in primary care facilities was set low deliberately by limiting the provision of medicines to the range of the essential medicines list and imposing a zero-markup policy for sales of medicines. Meanwhile, patients could enjoy a higher percentage of insurance reimbursement compared with similar services delivered in the hospitals [12].

In recent years, income disparities in China are widening. This has raised some worries about the emergence of a two-tier health care delivery system: a cheaper one (GP) for the poor and a more expensive one (hospitals) for the rich. This is because the choice of the poor is much more sensitive to price signal than that of the rich [1, 4]. Some studies in the European countries have proved that income-related inequities are indeed more likely to appear in specialist care and hospital services than in GP services [15, 16]. But there is a paucity in the literature documenting inequalities in GP and specialist services in China.

This study aimed to measure inequalities in GP and specialist services in China and decompose the contributions of associated factors to these inequalities. The findings will not only shed some light on the current status of the two service delivery systems, but also provide some insight into the potential drawbacks of relevant policies.

## Methods

### Study setting

This study was conducted in Hangzhou, the capital city of Zhejiang province, an economically developed region located in the southeast coast of China. Hangzhou has a population of about nine million residing on a land of 16,596 km^2^. Like in other regions of China, primary care services are mainly delivered by publicly-owned community health facilities in Hangzhou. In 2017, Hangzhou had 1363 CHCs and stations (1275 in urban areas) employing 16,159 (12%) health workers, compared with 302 hospitals employing 92,621 (69%) health workers. The majority of GPs worked in CHCs. Enrollees of the urban and rural resident health insurance programs in Hangzhou were entitled with 70% reimbursement of medical bills for a visit to CHCs, compared with 40% for a visit to specialists in a tertiary hospital.

### Data collection

A cross-sectional questionnaire survey was conducted on urban community residents in Hangzhou from 1 July 2017 to 31 August 2017. We adopted a multistage sampling strategy to select study participants. The first stage involved a selection of five (Jianggan, Xiacheng, Gongshu, Xihu and Shangcheng) out of the ten urban districts of Hangzhou, considering diversities in geographical location and economy development. In the second stage, two communities (one large and one small) were identified in each selected district. Finally, we randomly selected households in each sampled community based on the house number and two trained investigators were dispatched to invite the residents in selected households. Those who were 18 years or older, lived in the sampled communities for at least six months, and were able to communicate and provide informed consent were eligible to participate in the study. The survey was completely voluntary. The participants were requested to read the informed consent letter and gave oral consent prior to the survey. The questionnaire was administered through face-to-face interviews.

According to selected community size, we distributed 180 to 220 questionnaires to each sample unit. A total of 2000 residents were invited to participate in the survey and 1485 (74%) accepted the invitation. This resulted in a final sample size of 1048 for statistical analyses after exclusion of the returned questionnaires containing logic errors or missing values on key variables, indicating an effective response rate of 71%. This sample size is large enough to enable decomposition analysis on the 11 contributors of the inequality in access to GP and specialist care [17, 18].

### Outcome variables

Two indicators were calculated to measure the probability and frequency of the use of GP and specialist services, respectively. For probability, the respondents were asked whether they visited GP and specialist over the past four weeks prior to the survey (Additional file 1). Respondents needed to answer yes (code = 1) or no (code = 0). If yes, the respondents were asked to estimate how many times they visited a GP (in CHCs) and a specialist (in hospitals) over the past four weeks prior to the survey. Some CHCs also provided specialist consultations. But the number was small and was not included in the calculation of the two indicators. The probability indicator reflects the percentage of respondents seeking medical attention from GPs or specialists; whereas, the frequency indicator reflects the total number of visits to GPs or specialists in those who sought medical consultations [1, 19].

### Independent variables

We followed the Andersen’s social behavioral model in the selection of independent variables [1, 20]. These variables were categorised into three groups: predisposing, enabling and need factors. The predisposing factor included gender and age. The enabling factor represented barriers and facilitators for access to health care, such as household registration (local vs non-local), marital status (single, married, divorced/separated/widowed), income, years of education, health insurance coverage, and health resources available. The need factor was reflected by self-rated health and chronic conditions.

In this study, income was divided into five levels according to the per capita monthly household income of the respondents, ranging from below 3000 Yuan to equal or higher than 10,000 Yuan. Health resources were measured by the availability of a contracted GP and the walking distance to the nearest CHC. Chronic illness was defined as a condition diagnosed by a physician and lasted over the past six months [19–21]. Self-rated health was measured using a three-point Likert scale (poor, fair, good).

### Statistical analysis

Concentration index (C) was employed to measure inequalities in the use of GP and specialist services. It quantified the degree of income-related inequality with a range between − 1 and + 1 [22]. A negative C value indicates a pro-poor effect with services being more concentrated on the poor, and vice versa. A zero C value indicates an absent of inequality.

$$ \mathrm{C}=\frac{2}{\mu } COV\left(y,\gamma \right) $$

Where C was defined in terms of the covariance between the outcome variable (*y*) and the fractional ranks of household income (*γ*); μ is the mean of *y*.

We established regression models on the outcome variables (*y*) as proposed by Wagstaff and colleagues to decompose the contributions of independent variables to the inequality [23, 24].

$$ {y}_i={a}^m+{\sum}_k{\beta}_k^m{x}_{ki}+{\mu}_i $$

Where ${\beta}_k^m$ is the marginal effect (dy/dx) of each x; *μ*_*i*_ indicates the error term generated by the regression model. In our study, the logistic regression for probability of GP and specialist visits and zero-truncated Poisson regression for frequency of GP and specialist visits (frequency is more than zero) were established to analyse relationship between the outcome variables and various independent variables [19, 25, 26].

Then, the concentration index for y can be written as:

$$ \mathrm{C}={\sum}_k\left({\beta}_k\overline{x_k}/\mu \right){c}_k+{GC}_{\varepsilon }/\mu $$

Where C is the concentration index of health service utilization; *β*_*k*_ is the marginal effect of *x*_*k*_; $\overline{x_k}$ and *c*_*k*_ are the mean and the concentration index of *x*_*k*_; μ is the mean of y; *GC*_*ε*_ is the generalised concentration index for ε. This equation shows that the total concentration index is made up of two components: explained component and residual component.

The contribution of each independent variable to the inequity was presented as an absolute value and a percentage value.

## Results

### Characteristics of respondents

Most (60.00%) respondents were women and 45.8% were in the age between 26 and 35. The majority (71.40%) were married. Slightly more than half (54.00%) of the respondents held a household registration with the local community. The respondents were well educated, with 61.7% having a university degree. More than 90% of the respondents were covered by social health insurance. Overall, the respondents were healthy: 19.10% reported a chronic condition and 7.20% rated poor health.

A total of 427 (40.74%) and 365 (34.82%) respondents reported a visit to GPs and specialists over the past four weeks, respectively. These included 168 (16.03%) respondents who visited both GPs and specialists. Of those who sought medical consultations, on average, they visited 2.10 times to GPs and 1.66 times to specialists (Table 1).

**Table 1:** Characteristics of respondents

characteristics of respondents	Number	(%)	Visit to GPs(*n* = 427)		Visit to Specialists(*n* = 365)	
			(%)	Frequency	(%)	Frequency
Total			40.74	2.10 ± 1.73	34.82	1.66 ± 0.92
Gender						
Male	419	40.00	40.2	2.37 ± 2.02	41.4	1.70 ± 0.878
Female	629	60.00	59.8	1.91 ± 1.48	58.6	1.64 ± 0.963
Age (years)						
18–25	216	20.60	13.1	1.54 ± 1.15	15.3	1.38 ± 0.64
26–35	480	45.80	46.3	2.04 ± 1.72	44.7	1.75 ± 0.96
36–45	175	16.70	14.5	2.11 ± 1.41	21.4	1.78 ± 0.98
46–55	100	9.50	12.1	2.13 ± 1.31	10.1	1.35 ± 0.58
≥ 56	77	7.30	14.0	2.77 ± 2.48	8.5	1.77 ± 1.17
Household registration						
Non-local	482	46.00	42.3	1.73 ± 1.21	46.0	1.60 ± 0.87
Local	566	54.00	57.7	2.36 ± 1.99	54.0	1.72 ± 0.97
Marital status						
Single	237	22.60	15.7	2.24 ± 2.46	15.6	1.58 ± 0.80
Married	748	71.40	75.5	2.131 ± .61	76.2	1.71 ± 0.98
Divorced/separated/widowed	63	6.00	8.9	1.58 ± 0.94	8.2	1.40 ± 0.49
Per Capita Monthly household income (¥)						
< 3000	60	5.70	4.9	1.57 ± 1.02	2.2	1.38 ± 0.51
3000-	278	26.50	29.4	2.292 ± .03	23.8	1.74 ± 1.00
5000-	252	24.00	24.3	2.201 ± .91	19.5	1.65 ± 1.03
8000-	168	16.00	14.7	1.89 ± 1.19	16.7	1.48 ± 0.69
≥ 10,000	288	27.50	26.6	2.01 ± 1.53	37.8	1.72 ± 0.92
Education						
Primary school or below	37	3.50	6.5	2.11 ± 1.10	5.2	1.16 ± 0.37
Junior high school	99	9.40	11.9	2.45 ± 2.62	5.8	1.24 ± 0.70
Senior high school	265	25.30	26.9	2.04 ± 1.36	23.3	1.85 ± 1.05
Bachelor degree	434	41.40	37.6	2.11 ± 1.76	41.6	1.55 ± 0.89
Postgraduate degree	213	20.30	17.1	1.921 ± .61	24.1	1.89 ± 0.90
Medical insurance						
Uninsured	100	9.50	5.6	1.96 ± 1.16	5.5	1.85 ± 0.67
Insured	948	90.50	94.4	2.111 ± .76	94.5	1.65 ± 0.94
Contracted GP						
No	706	67.40	50.5	2.101 ± .86	58.9	1.64 ± 0.92
Yes	342	32.60	49.5	2.091 ± .59	41.1	1.69 ± 0.94
Walking distance of nearest CHC (minutes)						
<15	378	36.10	38.6	2.24 ± 1.78	37.8	1.67 ± 0.97
15–30	403	38.50	35.7	1.81 ± 1.30	30.7	1.58 ± 0.84
>30	267	25.50	25.7	2.28 ± 2.10	31.5	1.73 ± 0.94
Chronic diseases						
No	879	83.90	73.6	2.03 ± 1.67	73.2	1.62 ± 0.86
Yes	169	16.10	26.4	2.30 ± 1.89	26.8	1.79 ± 1.07
Self-rated health status						
Poor	75	7.20	12.1	2.54 ± 2.04	12.0	2.11 ± 1.22
Fair	489	46.70	46.0	2.05 ± 1.74	48.8	1.55 ± 0.87
Good	484	46.20	41.8	2.03 ± 1.61	39.2	1.66 ± 0.84

### Inequalities in the use of GP and specialist services

A negative concentration index was found for both probability (− 0.0464) and frequency (− 0.1346) of GP visits, indicating a pro-poor effect (*p* < 0.05). The poor people were more likely to visit a GP and visited GPs more frequently than their rich counterparts.

A positive concentration index was found for both probability (0.1258) and frequency (0.1279) of visits to specialists, indicating a pro-rich effect (*p* < 0.05). The rich people were more likely to visit a specialist and visited specialists more frequently than their poor counterparts.

### Decomposition of inequalities in the use of GP and specialist services

**Fig. 1:**
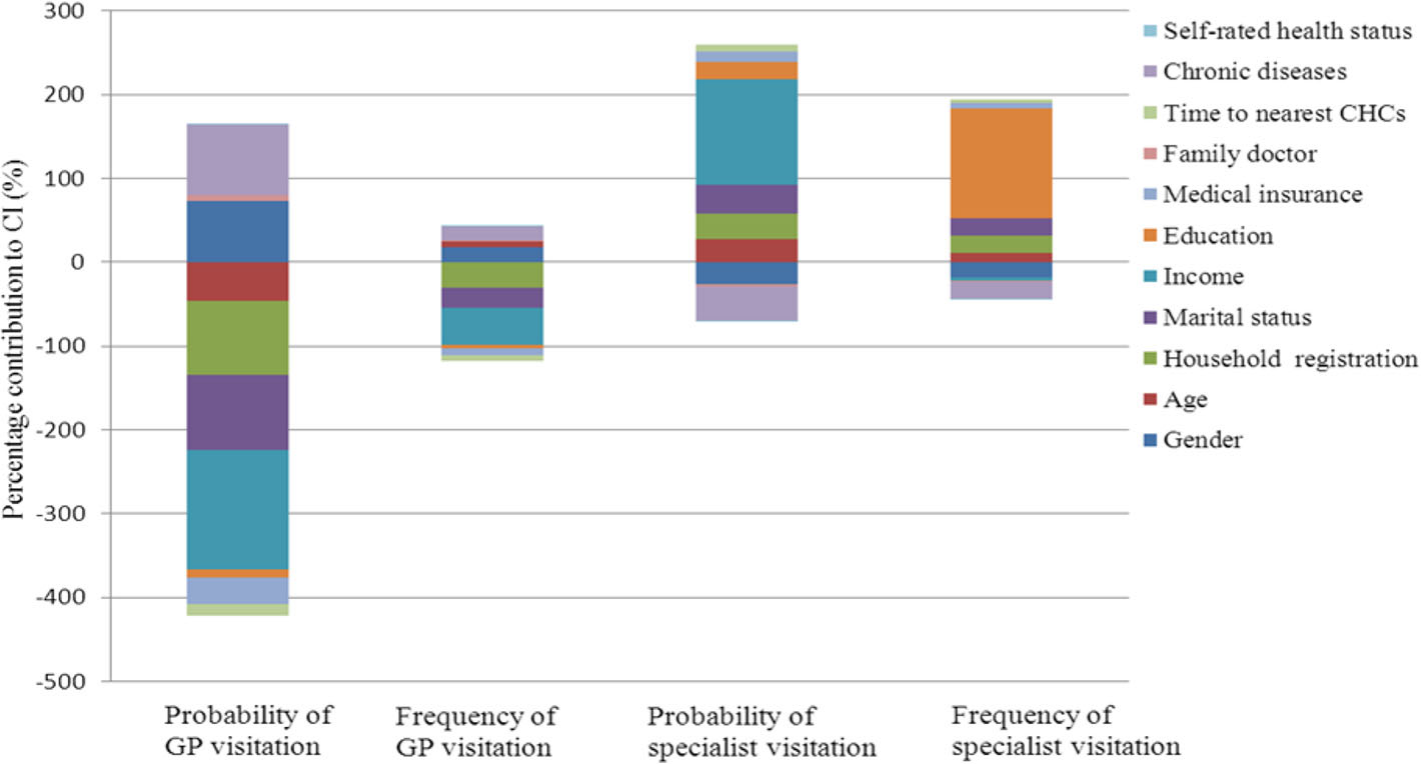
Contributions of different variables to inequalities of GP and specialist services utilization

The enabling factor made a significant contribution to the inequality in the use of GP services after adjustment for variations in predisposing and need factors (Table 2). Income was the biggest contributor to the pro-poor distribution of the probability (− 142.35%) and frequency (− 44.18%) of visits to GPs, followed by household registration and marital status (Fig. 1). The poor and those who were not local and not married were more likely to visit GPs and did so more frequently.

**Table 2:** Decomposition of concentration index of probability and frequency of GP visits

variables	C_k_	Probability of GP visits (*n = 1048*)			Frequency of GP visits (*n = 427*)		
		Margin	Absolute Contribution	Percentage contribution	Margin	Absolute Contribution	Percentage contribution
Gender							
Male	Ref.				Ref.		
Female	−0.0568	0.4081	−0.0339	73.11	1.4663*	−0.0238	17.68
Age (years)							
18–25	Ref.				Ref.		
26–35	0.1422	0.4434	0.0704	− 151.80	2.6450*	0.0820	−60.94
36–45	0.1159	0.4088	0.0193	−41.59	3.8130*	0.0351	−26.11
46–55	− 0.2663	0.4815	− 0.0298	64.30	4.9047*	−0.0593	44.08
≥ 56	−0.3595	0.5967*	−0.0385	82.88	5.3661**	−0.0675	50.16
Household registration							
Non-local	Ref.				Ref.		
Local	0.0789	0.3919	0.0407	−87.79	2.0609**	0.0418	−31.07
Marital status							
Single	Ref.				Ref.		
Married	0.0613	0.4114	0.0439	−94.61	1.5058*	0.0314	−23.31
Divorced/separated/widowed	−0.0338	0.4469	−0.0022	4.77	0.4769**	− 0.0005	0.34
Per Capita Monthly household income (¥)							
< 3000	Ref.						
3000-	−0.6164	0.5394*	−0.2151	463.67	3.1450**	−0.2449	181.95
5000-	−0.1106	0.5343*	−0.0347	74.71	3.1884*	−0.0404	30.00
8000-	0.2900	0.5479*	0.0621	− 133.88	2.7689	0.0613	−45.54
≥ 10,000	0.7137	0.5268*	0.2537	− 546.85	3.0143*	0.2835	−210.59
Education							
Primary school or below	Ref.				Ref.		
Junior high school	−0.4104	0.2543*	− 0.0241	51.84	1.4089	−0.0260	19.33
Senior high school	−0.1018	0.2804*	−0.0176	37.95	1.5476	−0.0190	14.10
Bachelor degree	0.0373	0.2830*	0.0107	−22.98	1.6335	0.0120	−8.93
Postgraduate degree	0.3241	0.2238*	0.0359	−77.47	1.2210	0.0383	−28.45
Medical insurance							
Uninsured	Ref.				Ref.		
Insured	0.0158	0.4198*	0.0146	−31.54	1.6416	0.0112	−8.30
Contracted GP							
No	Ref.				Ref.		
Yes	−0.0074	0.5864**	−0.0035	7.44	1.5024	− 0.0017	1.28
Walking distance of nearest CHC (minutes)							
<15	Ref.				Ref.		
15–30	−0.0347	0.4071	−0.0132	28.55	1.4540	−0.0092	6.86
>30	0.0770	0.4085	0.0195	−42.13	1.9488	0.0182	−13.53
Chronic diseases							
No	Ref.				Ref.		
Yes	−0.1985	0.5013*	−0.0388	83.69	1.4941	−0.0226	16.79
Self-rated health status							
Bad	Ref.				Ref.		
Fair	−0.0209	0.3230*	−0.0077	16.65	1.5555	−0.0072	5.37
good	0.0196	0.3106*	0.0069	−14.78	1.5650	0.0067	−5.01
LR chi2		181.57**			79.80**		
R^2^		0.1281			0.1207		

Note:*:*p* < 0.05, * *: *p* < 0.001

Similarly, the enabling factor also made a significant contribution to the inequality in the use of specialist services after adjustment for variations in predisposing and need factors (Table 3). Income remained the biggest contributor (125.91%) to the pro-rich distribution of the probability of specialist visits, followed by marital status (34.57%) and household registration (30.36%, Fig. 1). The wealthier people and those who were married and local were more likely to visit specialists. However, income was no longer a big contributor to the unequal distribution of the frequency of specialist visits. The pro-rich distribution of the frequency of specialist visits was mainly shaped by education (131.22%), household registration (21.07%) and marital status (21.47%, Fig. 1). Those who were better educated, local and married visited specialists more frequently.

**Table 3:** Decomposition of concentration index of probability and frequency of specialist visitation

variables	C_j_	Probability of specialist visits (*n = 1048*)			Frequency of specialist visits (*n = 365*)		
		Margin	Absolute Contribution	Percentage contribution	Margin	Absolute Contributio*n*	Percentage contribution
Gender							
Male	Ref.				Ref.		
Female	−0.0568	0.3465	−0.0337	−26.82	1.1044	−0.0225	−17.62
Age (years)							
18–25	Ref.				Ref.		
26–35	0.1422	0.3148	0.0571	45.41	1.4444*	0.0549	42.95
36–45	0.1159	0.3525	0.0203	16.12	1.6410	0.0198	15.47
46–55	−0.2663	0.3049	−0.0224	−17.78	1.0627	−0.0163	−12.77
≥ 56	−0.3595	0.2498	−0.0203	−16.15	2.6640*	−0.0454	−35.51
Household registration							
Non-local	Ref.				Ref.		
Local	0.0789	0.3322	0.0382	30.36	1.1183	0.0269	21.07
Marital status							
Single	Ref.				Ref.		
Married	0.0613	0.3701	0.0463	36.83	1.1000	0.0289	22.56
Divorced/separated/widowed	−0.0338	0.4538	−0.0028	−2.26	1.0574	−0.0014	−1.09
Per Capita Monthly household income (¥)							
< 3000	Ref.				Ref.		
3000-	−0.6164	0.5995**	−0.2670	− 212.25	1.8126	− 0.1692	−132.29
5000-	−0.1106	0.5671*	−0.0434	−34.53	1.4094	−0.0226	−17.69
8000-	0.2900	0.6627**	0.0859	68.31	1.0810	0.0294	22.97
≥ 10,000	0.7137	0.6626**	0.3829	304.38	1.2922	0.1565	122.37
Education							
Primary school or below	Ref.				Ref.		
Junior high school	−0.4104	0.1499*	−0.0163	−12.94	2.2946	−0.0522	−40.83
Senior high school	−0.1018	0.2491	−0.0178	−14.18	8.0541**	−0.1209	−94.51
Bachelor degree	0.0373	0.2772	0.0128	10.20	5.5061**	0.0534	41.75
Postgraduate degree	0.3241	0.2663	0.0473	37.60	7.7246**	0.2875	224.81
Medical insurance							
Uninsured	Ref.				Ref.		
Insured	0.0158	0.3585*	0.0146	11.58	1.0551*	0.0090	7.02
Contracted GP							
No	Ref.				Ref.		
Yes	−0.0074	0.4107*	−0.0029	−2.28	1.1306	−0.0017	−1.29
Walking distance of nearest CHC (minutes)							
<15	Ref.				Ref.		
15–30	−0.0347	0.3165	−0.0114	−9.09	0.9143	−0.0069	−5.41
>30	0.0770	0.3888	0.0226	17.97	1.0282	0.0125	9.80
Chronic diseases							
No	Ref.				Ref.		
Yes	−0.1985	0.4747*	−0.0512	−40.66	1.1189	−0.0253	−19.76
Self-rated health status							
Bad	Ref.				Ref.		
Fair	−0.0209	0.2964*	−0.0085	−6.73	0.7981*	−0.0048	−3.73
good	0.0196	0.2655*	0.0067	5.29	0.8222*	0.0043	3.38
LR chi2		129.40**			119.03**		
R^2^		0.0955			0.1464		

Note:*: *p* < 0.05, * *: *p* < 0.001

## Discussion

This study proved that there exist inequalities in the use of GP and specialist services in Hangzhou China. The distribution of GP services tends to bias toward the poor; whereas the distribution of specialist services tends to bias toward the rich. These results are consistent with findings of studies conducted in some other countries [15, 19, 27]. Understandably, poor people are more sensitive to price signals than their rich counterparts. Without a referral system put in place in China, the rich are more likely to bypass the cheaper GP services for the more expensive specialist services [28]. By contrast, the lower pricing level and higher insurance compensation rates for GP services are more attractive to those who have limited capacity to pay for medical care [1].

The emergence of the two systems, one for the poor and another for the rich, is concerning. For a long time, there has been a big gap in high-quality health resources between CHCs and hospitals in China, which resulted in a belief that the quality of specialist services provided in the hospital setting is higher than that of GP services provided in CHCs [29]. Therefore, inequalities in the use of GP and specialist services could have a significant impact on health care disparities between the rich and the poor.

Similar to previous studies undertaken elsewhere [15, 19], income is the biggest contributor of the inequalities in the use of GP and specialist services. Although China has established an almost universal health insurance system (more than 95% coverage), there are some obstacles to discourage the poor from obtaining benefits from medical insurance fairly compared with the rich. For example, not all medical expenses can be reimbursed and residents covered by medical insurance have to burden high amount of out-of-pocket costs [30]. Thus, patients with low income are more likely to visit affordable GP services. But the rich are the opposite. This study found very limited contribution of health insurance to inequality.

In this study, education was identified as the biggest contributor to the inequality in the frequency of visits to specialists. People with a higher education level used higher-priced specialist services significantly more often. People with higher levels of education tend to have higher expectations for their own health [31]. Also, previous studies suggested that people with high-level education tend to have a higher critical requirement on quality and more knowledge of health services [32, 33]. In addition to the distrust on community health services, these people prefer specialists in hospitals in the hope of receiving better services.

Social support may play a role in the inequality. Those who are local and married are likely to enjoy higher family and social support, resulting in a higher likelihood and intensity of more prestigious specialist services. People with high social or family support have higher requirements for quality of health services [34, 35]. In addition, the local can benefit more from insurance than their non-local counterparts, enjoying convenient and higher proportion of reimbursements. Thus, these factors have an effect on inequalities of specialists services.

The contributions of income, education and social support remained to be significant after controlling for the influence of the predisposing and need factors. For example, older people are more likely to visit a GP than the younger ones [36]. People with chronic conditions are more likely to visit GPs [16, 37].

It is worth noting that signing a contract with GPs contributes little to the inequality of GP services. This result is inconsistent with previous findings [38]. A potential reason is that the GP system in China is in the initial development stage. A serious shortage of GPs means that one GP has to sign contracts with 2000 residents or even more. Many contracted residents may not be able to receive the corresponding services. In addition, patients still enjoy freedom to choose medical providers even with a contract. Therefore, signing a contract with GP did not create enough effect on the preference of patients.

With the implementation of the tiered referral policy in China, the government encourages patients to make a rational choice on different levels of health care in line with their needs. However, this study suggests that choice of health services are affected by socioeconomic factors which can result in income-related inequality in health services. Therefore, appropriate policy and intervention strategies should be implemented to reduce these inequalities. Firstly, considering the greatest contribution of income and education to inequality, income redistributing measures is a feasible way to reduce the inequality, especially in pro-rich inequality of specialist visits. Increasing investment in education can also be considered. Secondly, more welfare benefits should be provided for the disadvantaged people (e.g. the elderly and those with low income and chronic diseases, etc.), consequently reducing barriers for them to use specialist services in hospitals. Thirdly, a referral system based on needs rather than the ability to pay should be established, thus leaving less room for inequalities in the utilization of specialist care to occur. This will require some fundamental changes in the infrastructure, including resource allocations (control of hospitals), insurance arrangements (reducing barriers in access to health care at all levels) and culture shifts (trust, waiting list, and referral) [39].

### Limitation

There are several limitations to this study which should be mentioned. Firstly, all of the data employed in our study were self-reported by residents, which could result in recall bias especially in frequency of GP and specialist visits. Secondly, the sample involved only Hangzhou city in China, and the proportion of the elderly population in the sample is slightly smaller possibly due to selection bias in the survey. Generalisation of the findings should be done cautiously. Thirdly, only two variables (probability and frequency of visis) were used to measure GP and specialist services.

## Conclusion

Strong income-related inequalities exist in GP and specialist services in Hangzhou China. Among the factors associated with these inequalities, income and education make the greatest contribution. Therefore, reducing disparities in socioeconomic status of people should be considered as an effective intervention strategy. In addition, other factors, such as age, marital status, and chronic conditions also affect inequality of GP and specialist services. Some preferential policy and intervention strategies associated with these factors should be taken into account.

## Additional file

Additional file 1: Qusetionnaire. (DOCX 19 kb)

## Abbreviations

C: Concentration index
CHCs: Community health centers
GPs: General practitioners

## References

[1] Zhou Z, Gao J, Fox A (2011). Measuring the equity of inpatient utilization in Chinese rural areas. BMC Health Serv Res.

[2] Gao J, Gao J, Tang S (2001). changing access to health services in urban China: implications for equity. Health Policy Plan.

[3] Fang P, Dong S, Xiao J, Liu C, Feng X, Wang Y (2010). regional inequality in health and its determinants: evidence from China. Health Policy.

[4] Gao J, Qian JC, Tang SL, Eriksson B, Blas E (2002). health equity in transition from planned to market economy in China. Health Policy Plan.

[5] Wang Y, Wang J, Maitland E (2012). Growing old before growing rich: inequality in health service utilization among the mid-aged and elderly in Gansu and Zhejiang provinces, China. BMC Health Serv Res.

[6] Luo J, Zhang X, Jin C (2009). inequality of access to health care among the urban elderly in northwestern China. Health Policy.

[7] Chen M, Chen W, Zhao Y (2012). New evidence on financing equity in China's health care reform: a case study on Gansu province China. BMC Health Serv Res.

[8] Xinyu Z, Lin Z, Cui Z (2015). Study on equity and efficiency of health resources and services based on key indicators in China. PLoS One.

[9] Zhu D, Guo N, Wang J, Nicholas S, Chen L (2017). Socioeconomic inequalities of outpatient and inpatient service utilization in China: personal and regional perspectives. Int J Equity Health.

[10] Zhang T, Xu Y, Ren J (2017). Inequality in the distribution of health resources and health services in China: hospitals versus primary care institutions. Int J Equity Health.

[11] Tang Liyang (2013). The Chinese community patient’s life satisfaction, assessment of community medical service, and trust in community health delivery system. Health and Quality of Life Outcomes.

[12] Zhao Y, Lin J, Qiu Y (2017). Demand and signing of general practitioner contract service among the urban elderly: a population-based analysis in Zhejiang Province, China. Int J Environ Res Public Health.

[13] Liu Q, Wang B, Kong Y (2011). China’s primary health-care reform. Lancet.

[14] Kong X, Yang Y (2015). The current status and challenges of community general practitioner system building in China. Q J Med.

[15] Stirbu I, Kunst AE, Mielck A (2011). Inequalities in utilization of general practitioner and specialist services in 9 European countries. BMC Health Serv Res.

[16] Masseria C, Giannoni M (2010). Equity in access to health care in Italy: a disease-based approach. Eur J Pub Health.

[17] Peduzzi P, Concato J, Feinstein A (2007). Importance of events per independent variable in proportional hazards regression analysis. II. Accuracy and precision of regression estimates. J Clin Epidemiol.

[18] Peduzzi Peter, Concato John, Kemper Elizabeth, Holford Theodore R., Feinstein Alvan R. (1996). A simulation study of the number of events per variable in logistic regression analysis. Journal of Clinical Epidemiology.

[19] Asada Y, Kephart G (2007). Equity in health services use and intensity of use in Canada. BMC Health Serv Res.

[20] Andersen R, Newman JF (1973). Societal and individual determinants of medical care utilization in the United States. Milbank Q.

[21] Gravelle H, Sutton M, Morris S (2003). Modeling supply and demand influences on the use of health care: implications for deriving a needs-based capitation formula. Health Econ.

[22] Wagstaff A, Paci P, Van Doorslaer E (1991). On the measurement of inequalities in health. Soc Sci Med.

[23] O’Donnell O, Van Doorslaer E, Wagstaff A (2008). Analyzing health equity using household survey data.

[24] Wagstaff A, van Doorslaer E, Watanabe N (2003). On decomposing the causes of health sector inequalities, with an application to malnutrition inequalities in Vietnam. J Econ.

[25] Xie T, Aickin M (1997). A truncated poisson regression model with applications to occurrence of adenomatous polyps. Stat Med.

[26] Tucker JS, Hu J, Golinelli D (2012). Social network and individual correlates of sexual risk behavior among homeless MSM youth. J Adolesc Health.

[27] Lostao L, Blane D, Gimeno D (2014). Socioeconomic patterns in use of private and public health services in Spain and Britain: implications for equity in health care. Health &Place.

[28] Pan X, Dib HH, Wang X (2006). Service utilization in community health centers in China: a comparison analysis with local hospitals. BMC Health Serv Res.

[29] Wanga H, Gusmano MK, Cao Q (2011). An evaluation of the policy on community health organizations in China: will the priority of new healthcare reform in China be a success?. Health Policy.

[30] Liu J, Shi L, Meng Q, Khan MM (2012). Income-related inequality in health insurance coverage: analysis of China health and nutrition survey of 2006 and 2009. Int J Equity Health.

[31] Regidor E, Martínez D, Calle ME (2008). Socioeconomic patterns in the use of public and private health services and equity in health care. BMC Health Serv Res.

[32] Henbest RJ, Stewart M (1990). Patient-centredness in the consultation. 2: does it really make a difference?. Fam Pract.

[33] Blackwell DL, Martinez ME, Gentleman JF, Sanmartin C, JM B (2009). Socioeconomic status and utilization of health care services in Canada and the United States: findings from a binational health survey. Med Care.

[34] Sellars B, Garza MA, Fryer CS, Thomas SB (2010). Utilization of health care services and willingness to participate in future medical research: the role of race and social support. J Natl Med Assoc.

[35] Peng TR, Navaie-Waliser M, Feldman PH (2003). Social support, home health service use, and outcomes among four racial-ethnic groups. The Gerontologist.

[36] Choi, G N: Changes in labor force activities and income of the elderly before and after retirement: a longitudinal analysis**.** J Sociol Soc Welf 1994, 21**:**5.

[37] Alkhawaldeh A, Holm MB, Qaddumi J (2014). A cross-sectional study to examine factors associated with primary health care service utilization among older adults in the Irbid governorate of Jordan. Current Gerontology and Geriatrics Research.

[38] Fung CSC, Wong CKH, Fong DY (2015). Having a family doctor was associated with lower utilization of hospital-based health services. BMC Health Serv Res.

[39] Forrest CB, Nutting PA, Von Schrader S (2006). Primary care physician specialty referral decision making: patient, physician, and health care system determinants. Med Decis Mak.

