# Supplementary material for: General practice for the poor and specialist services for the rich: inequality evidence from a cross-sectional survey on Hangzhou residents, China
Source: Int J Equity Health. 2019 May 14;18:69. doi: 10.1186/s12939-019-0966-6 (PMC6518799; doi:10.1186/s12939-019-0966-6)
Supplement: Supplementary file 1 — Qusetionnaire. (DOCX 19 kb) [file 12939_2019_966_MOESM1_ESM.docx]

**QUSETIONNAIRE**

**1. Your gender:** (1) Male (2) Female

**2. Your age:** _____________ years old

**3. Your household registration:**

(1) Hangzhou household registration (2) Non-Hangzhou household registration

**4. Your education level:**

(1) Elementary school and below

(2) Junior high school

(3) Technical secondary school / high school

(4) Junior College/Undergraduate

(5) Bachelor degree or above

**5. Monthly income per capita of your family:**

(1) 3000 or less

(2) 3000-5000

(3) 5000-8000

(4) 8000-10000

(5) 10000 or more

**6. Your marital status:**

(1) Unmarried

(2) Married

(3) Divorced/separated/widowed

**7.Are you covered by health insurance?**

(1) Yes (2) No

**8. Do you have chronic diseases diagnosed by doctors, such as high blood pressure, diabetes, etc?**

(1) Yes (2) No

**9. In the past six months, how do you feel about your health?**

(1) Bad (2) Fair (3) Good

**10. How long does it take to walk from your home to the nearest community health service?**

(1) less than 15 minutes (2) 15-30 minutes (3) 30 minutes or more

**11. Have you signed up with general practitioner in a community health service?**

(1) Yes (2) No

**12. Have you visited a general practitioner in the community health service in the past 4 weeks?**

(1) Yes (2) No (IF NO, skip the 13th question)

**13. How many times you visited a general practitioner over the past four weeks?** __________times

**14. Have you visited a specialist in hospitals in the past 4 weeks?**

(1) Yes (2) No (IF NO, skip the 15th question)

**15. How many times you visited a specialist in hospitals over the past four weeks?** __________times
